# Facile Synthesis of Antimony Tungstate Nanosheets as Anodes for Lithium-Ion Batteries

**DOI:** 10.3390/nano9121689

**Published:** 2019-11-25

**Authors:** Yong Liu, Yue Wang, Fei Wang, Zhenxiao Lei, Wanhong Zhang, Kunming Pan, Jing Liu, Min Chen, Guangxin Wang, Fengzhang Ren, Shizhong Wei

**Affiliations:** 1 Collaborative Innovation Center of Nonferrous Metals of Henan Province, Henan Key Laboratory of Non-Ferrous Materials Science & Processing Technology, School of Materials Science and Engineering, Henan University of Science and Technology, Luoyang 471023, China; 18438616683@163.com (Y.W.); 15737937208@163.com (F.W.); 151402090112@stu.haust.edu.cn (Z.L.); zhangwh@haust.edu.cn (W.Z.); 181414120220@stu.haust.edu.cn (J.L.); renfz@haust.edu.cn (F.R.); 2 National Joint Engineering Research Center for Abrasion Control and Molding of Metal Materials, Henan Key Laboratory of High-Temperature Structural and Functional Materials, Henan University of Science and Technology, Luoyang 471023, China; pankunming2008@163.com; 3 School of Agriculture, Henan University of Science and Technology, Luoyang 471023, China; 4 School of Materials Science and Energy Engineering, Foshan University, Foshan 528000, China; minchen1981@126.com

**Keywords:** antimony tungstate nanosheets, microwave hydrothermal method, lithium-ion batteries, anode, cycling performance, rate capability

## Abstract

Lithium-ion batteries (LIBs) have been widely used in the fields of smart phones, electric vehicles, and smart grids. With its opened Aurivillius structure, tungstate antimony oxide (Sb_2_WO_6_, SWO), constituted of {Sb_2_O_2_}^2n+^ and {WO_4_}^2n−^, is rarely investigated as an anode for lithium-ion batteries. In this work, Sb_2_WO_6_ with nanosheets morphology was successfully synthesized using a simple microwave hydrothermal method and systematically studied as an anode for lithium-ion batteries. The optimal SWO (SWO-60) exhibits a high specific discharge capacity and good rate capability. The good electrochemical performance could be ascribed to mesoporous nanosheets morphology, which is favorable for the penetration of the electrolyte and charge transportation. The results show that this nanostructured SWO is a promising anode material for LIBs.

## 1. Introduction

Lithium-ion batteries (LIBs) are widely used in the fields of portable devices, electric cars, and smart grids due to of their advantages, which include a higher working voltage and longer cycle life than many other energy storage systems [1,2,3,4,5,6,7,8,9]. However, the energy density of LIBs is still limited and may not meet demand, especially in the fields of high-performance electric vehicles and unmanned aerial vehicles, effectively hindering further development in these fields [10,11,12,13,14,15,16,17]. The reversible discharge capacity of commercial anodes (graphite), which is only ~372 mAh g^−1^, cannot meet the increasing demands for high-energy-density applications [18,19,20,21,22,23,24,25,26]. The use of proper anode materials with high capacities is a good strategy to solve the problem of LIBs [17]. Therefore, it is urgent that high-capacity negative materials for rechargeable lithium-ion batteries are developed and optimized.

As a potentially promising anode material for LIBs, transition metal oxides (TMO) have attracted broad attention due to their higher reversible capacity and proper redox voltage [25,26]. As one kind of TMO, oxysalts of tungsten, such as ZnWO_4_ [27], CaWO_4_ [28], FeWO_4_ [29], and Bi_2_WO_6_ [30,31], have been used as negative materials for lithium-ion batteries. For example, Zhang and colleagues [30] investigated Bi_2_WO_6_/graphene composite materials, which can exhibit 715.2 mAh g^−1^ at the first discharge at 50 mA g^−1^ and show excellent cycling performance. As a kind of tungstate, Sb_2_WO_6_ has a wide application in the field of photocatalysis [32,33,34] because of its unique layered structure and physicochemical properties [35,36]. Additionally, Sb_2_WO_6_ was recently reported as an anode for sodium-ion batteries with superior cycling and rate performance, delivering about 350 mA h g^−1^ after 100 cycles at 0.2 A g^−1^ [35]. Furthermore, the microwave hydrothermal method is well-known for effectively reducing reaction time and saving energy [37]. However, to the best of our knowledge, Sb_2_WO_6_ is rarely systematically investigated as an anode material for LIBs.

Herein, Sb_2_WO_6_ nanosheets were successfully fabricated through a microwave hydrothermal method, and their electrochemical performance was systematically studied as a negative electrode for LIBs. When used as a negative electrode material for LIBs, the optimal as-synthesized tungstate antimony oxide (SWO) sample exhibits a high reversible specific discharge capacity of ~698.95 mAh g^−1^ and remains at 424.22 mAh g^−1^ after 100 cycles at 0.2 A g^−1^. Furthermore, it exhibits a good rate performance.

## 2. Materials and Methods

### 2.1. Fabrication of Sb_2_WO_6_

All chemicals were directly used after purchase without further purification. As per the typical method, 0.1045 g Na_2_WO_4_·2H_2_O and 0.1446 g SbCl_3_ were separately dissolved in 20 mL and 10 mL of distilled water, respectively, and then stirred for 15 min. The Na_2_WO_4_·2H_2_O solution was added to the SbCl_3_ solution until a yellow suspension was formed, after which it was stirred for another 30 min. Next, the solution was added to a microwaveable water kettle (50 mL) and fixed in a microwave hydrothermal synthesizer (XH800, Beijing, China). A continuous microwave heating mode was set up to provide a stable heat source at a microwave power of 500 W. The temperature of the mixed solution quickly rose from room temperature to 160 °C and was maintained at 160 °C for different lengths of time (45 min, 60 min, and 75 min, which were denoted as SWO-45, SWO-60, and SWO-75, respectively). Afterwards, the resulting precipitates were filtered and washed several times with absolute ethanol and deionized water, and then the collected precipitation was dried overnight by lyophilization (LGJ-12, Beijing, China).

### 2.2. Material Characterization

Scanning electron micrographs were collected on a field-emission scanning electron microscope (FESEM, JSM-5610LV, JEOL, Akishima, Japan), and transmission electron micrographs and selected area electron diffraction patterns (SAED) were collected on a transmission electron microscope (TEM, JSM-2100F, 200 kV, Hitachinaka, Naka, Japan) to visually observe morphologies, particle sizes, etc. The crystallographic phase of the as-synthesized samples was characterized by X-ray diffraction (XRD, Bruker D8 ADVANCE, Cu kα source), and the 2θ-angle ranged from 10°–80°. Nitrogen adsorption-desorption isotherms were acquired by the Brunauer-Emmett-Teller (BET) method at 77 K from a Quadrasorb SI analyzer, and pore size distribution originated from the desorption branch based on the Barrett–Joyner–Halenda (BJH) theory.

### 2.3. Electrochemical Measurements

The electrochemical lithium storage performances were evaluated through a two-electrode system. Specifically, high-purity lithium foil (15.8 mm × 0.5 mm) was employed as the counter and reference electrode. After dissolving carboxymethylcellulose sodium (binder, CMC) in deionized water and stirring gently for 24 h to fabricate the working electrode, the slurry was made by mixing the Sb_2_WO_6_ samples, carbon black (Super P), and CMC at a weight ratio of 70:20:10. After fully stirring, the steady slurry was coated onto conductive copper foil and dried at 80 °C for 5 h. The dried electrodes were cut into a 12 mm-diameter disk to serve as a working electrode, and the active material loading in each disk was weighed to be 0.9–1.1 mg, corresponding to 0.8–0.97 mg cm^−2^. The batteries were assembled in the glove box with argon atmosphere. During assembly, the Celgard 2400 was used as a separator, while the electrolyte was made by dissolving 1M LiPF_6_ into a solvent consisting of ethylene carbonate (EC) and dimethyl carbonate (DMC) (EC/DMC, 1:1 in volume ratio). The galvanostatic discharge–charge measurements were performed on a LAND CT2001A in the voltage window from 0.01 to 3.0 V (vs. Li/Li^+^). An electrochemical impedance spectroscopy (EIS) was conducted ranging from 100 kHz to 0.01 Hz, and the cyclic voltammetry (CV) was measured in the range of 0.01–3.0 V (vs. Li/Li^+^) at 0.5 mV s^−1^. The two electrochemical tests discussed above were carried out on an electrochemistry workstation (CHI660E, Shanghai, China).

## 3. Results and Discussion

### 3.1. Structure and Morphology

**Figure 1:**
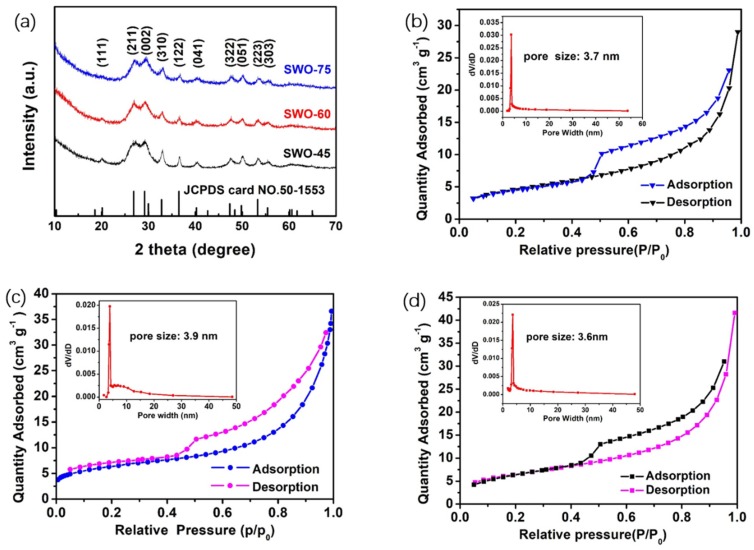
(**a**) X-ray diffractometry pattern of as-synthesized tungstate antimony oxide (SWO)-45, SWO-60, and SWO-75 samples. Nitrogen adsorption-desorption isotherms and pore-size distribution (inset) of (**b**) SWO-45, (**c**) SWO-60, and (**d**) SWO-75 samples.

Figure 1a illustrates the XRD patterns of the Sb_2_WO_6_ samples synthesized by a microwave hydrothermal method at different microwave heating times (45 min, 60 min, and 75 min). The peaks at 26.9° (211), 29.2° (002), 32.9° (310), and 36.6° (112) agree well with the diffraction pattern of Sb_2_WO_6_ (JCPDS card No.50-1553) [29]. Furthermore, no additional phase could be found, indicating that pure Sb_2_WO_6_ was successfully synthesized.

The Sb_2_WO_6_ samples synthesized by the different microwave heating times (SWO-45, SWO-60, and SWO-75) were also characterized by a nitrogen adsorption-desorption isotherms measurement. As depicted in Figure 1b–d, typical IV-type isotherms were obtained, indicating that almost no micropores existed in the Sb_2_WO_6_ samples [36]. A large number of mesopores (whose diameters are usually smaller than 30 nm) were observed in the pore-size distribution curves (inset in Figure 1b–d). The specific surface areas, mean pore sizes, and mean crystallite sizes of the three different SWO samples are listed in Table 1. According to the BET equation and the Barrett–Joyner–Halenda plot, the specific surface area of the SWO-60 sample was calculated as ~22.07 m^2^ g^−1^, and the mean pore size appeared at 3.9 nm. According to the Scherrer equation, the mean crystallite size was calculated as ~13.82 nm. In contrast, the specific surface area and the mean pore size of the SWO-45 sample was ~16.79 m^2^ g^−1^ and 3.7 nm, respectively. This specific surface area was much smaller than that of the SWO-60 sample, and the mean crystallite size of SWO-45 was higher than SWO-60. Although the specific surface area of the SWO-75 sample (~22.98 m^2^ g^−1^) was a little higher than the surface area of the SWO-60 sample, the main pore size was smaller than in the SWO-60 sample. It is believed that this mesoporous structure, with appropriate crystallite size, may be favorable for the transport and storage of lithium ions [38].

**Figure 2:**
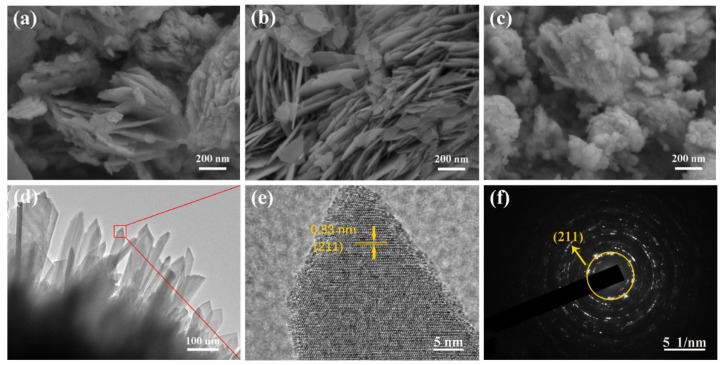
(**a**–**c**) Scanning electron microscope (SEM) images of as-prepared SWO-45, SWO-60, and SWO-75 samples; (**d**) transmission electron microscope (TEM) image of SWO-60; (**e**) high-resolution transmission electron microscope (HRTEM) image; and (**f**) selected area electron diffraction (SAED) pattern of the SWO-60 sample.

A scanning electron microscope and a transmission electron microscope were carried out to examine the microstructures and morphology of the SWO samples. As depicted in Figure 2a,b, the as-synthesized SWO-45 and SWO-60 samples exhibit similar nanosheet-like morphology. The nanosheets in SWO-45 (Figure 2a) are severely agglomerated and unevenly distributed, whereas the SWO-60 nanosheets are uniformly grown, and the thickness of the nanosheets is 12~21 nm, which might favor the charge transport and ion diffusion as well as the penetration of the electrolyte. In contrast, the SWO-75 sample (Figure 2c) seems to be of nanoparticle assembly, the reason for which may be that the microwave reaction time was too long, resulting in the overgrowth of nanosheets. As depicted in Figure 2d, the transmission electron microscope image further confirms flake morphology of the SWO-60 sample. A high-resolution transmission electron microscope (HRTEM) image is depicted in Figure 2e, with interplanar spacing of 3.3 Å in a single nanosheet, which corresponds to the spacing between the (211) crystal plane of the Sb_2_WO_6_. Figure 2f shows the SAED of SWO-60, indicating that the crystalline structure of SWO-60 is polycrystalline, which agrees well with the X-ray diffraction patterns depicted in Figure 1a.

**Figure 3:**
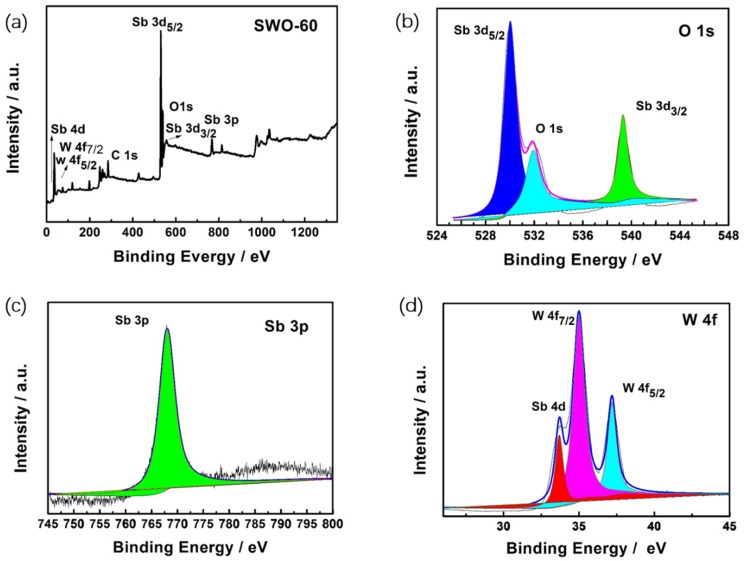
(**a**) X-ray photoelectron spectroscopy (XPS) survey of the SWO-60 sample, and the high-resolution XPS spectra of (**b**) Sb 3d and O 1s, (**c**) Sb 3p, and (**d**) Sb 4d and W 4f.

To further investigate the chemical composition and valence state of SWO-60, an X-ray photoelectron spectroscopy (XPS) was conducted. In Figure 3b, the binding energies of 530.9 eV and 540.3 eV are in accordance with the Sb 3d_5/2_ and Sb 3d_3/2_ with 3^+^ oxidation state, respectively [39]. In addition, the peak at 531.9 eV is the characteristic of O 1s. In Figure 3c, the peak at 767.9 eV could be attributed to the Sb 3p_3/2_ binding energy of Sb_2_WO_6_. Additionally, the peaks at 35.9 eV, 38.1 eV, and 34.5 eV are characteristics of W 4f_7/2_, W 4f_5/2, _ and Sb 4d, respectively, and W 4f_7/2_ and W 4f_5/2_ are in accordance with the oxidation state of W^6+^ [40,41].

### 3.2. Electrochemical Performance

**Figure 4:**
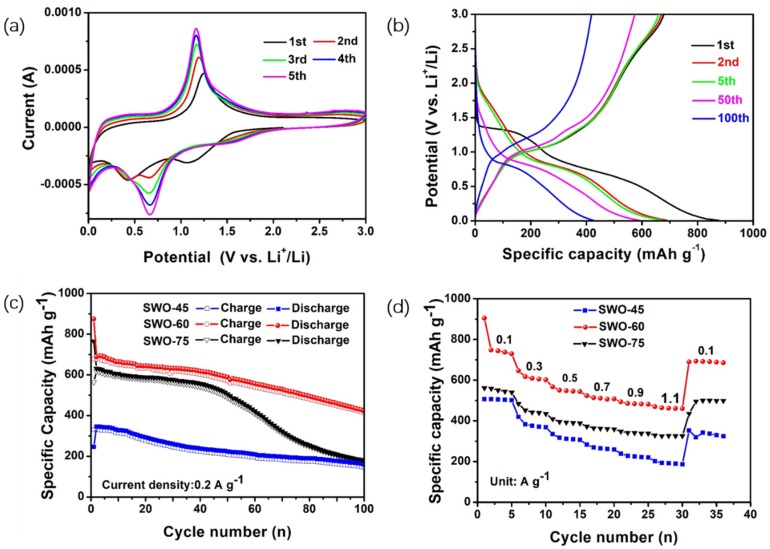
(**a**) Cyclic voltammetry curves of the SWO-60 electrode at 0.5 mV s^−1^; (**b**) cdischarge–charge profile of SWO-60 electrode at different cycles at 0.2 A g^−1^; (**c**) cycling performances of SWO-45, SWO-60, and SWO-60 electrodes at 0.2 A g^−1^; and (**d**) rate performance of SWO-45, SWO-60, and SWO-60 electrodes at current density ranging from 0.1–1.1 A g^−1^.

Electrochemical tests were conducted to examine the performances of different SWO samples in lithium-ion batteries (Figure 4). The cyclic voltammetry (CV) of a SWO-60 electrode at a scan rate of 0.5 mV s^−1^ between 0.01 V and 3 V is depicted in Figure 4a. The reduction peak located at ~1.125 V can be attributed to the formation of solid electrolyte interface (SEI) film, during which Sb_2_WO_6_ was decomposed and antimony oxide was converted into metallic antimony [35]. The reduction peak centered at ~0.43V could be attributed to the alloying-reaction that took place between Li and Sb, and the oxidation peak of 1.254V is related to the dealloying of Li_3_Sb [35,42]. As we can see, from the third cycle on, the cyclic voltammetry curves are well overlapped, indicating good reversibility of the SWO-60 electrode.

The discharge–charge profile of the SWO-60 electrode in the 1st, 2nd, 5th, 50th, and 100th cycles at 0.2 A g^−1^ is shown in Figure 4b. During the first cycle, the initial specific discharge and charge capacities of the Sb_2_WO_6_ electrode were ~875.06 and ~678.42 mAh g^−1^, respectively, corresponding to 77.52% in initial coulombic efficiency, which could be attributed to the formation of a solid electrolyte interphase during the first discharge process. As shown in Figure 4b, there are two well-defined plateaus at ~1.3 and ~0.7 V in the first discharge curve. The plateau of ~1.3 V disappears after the first cycle, while the ~0.7 V plateau remains in the following cycles, which is consistent with the CV discussed above. The cycling stabilities of different Sb_2_WO_6_ electrodes are shown in Figure 4c. The initial specific discharge capacities of the SWO-45, SWO-60, and SWO-75 electrodes are 246.03, 875.06, and 764.5 mAh g^−1^ at 0.2 A g^−1^, respectively. The discharge capacities of the SWO-45 and SWO-75 electrodes decrease rapidly, the specific discharge capacities of which undergo a decrease of ~49% and ~69%, respectively, from the 2nd to the 100th cycle, that is, they decrease at a rate of ~1.9 and ~4.4 mAh g^−1^ per cycle, respectively. In contrast, the reversible capacity of the SWO-60 electrode still delivers 424.22 mAh g^−1^ at 0.2 A g^−1^ after 100 cycles, corresponding to a decrease of ~39%, which is less than the other two electrodes, which may be associated with the uniform distributed nanosheets structure. However, as mentioned in the literature, the cycling stability of SWO-60 is inferior to Sb_2_WO_6_ for sodium-ion batteries [29], which may be attributed to the poor conductivity of SWO-60 [27], and its cycling stability could be improved through compositing with amorphous carbon, graphene, and other materials with higher conductivity, such as conducting polymers. Figure 4d illustrates the rate-performance of three electrodes at current densities ranging from 0.1 to 1.1 A g^−1^. The SWO-60 electrode displays reversible discharge capacities of 743.9, 513.7, and 462.2 mAh g^−1^ at 0.1, 0.7, and 1.1 A g^−1^, respectively. The SWO-60 electrode can still deliver 703.6 mA h g^−1^ when the current density reverts to 0.1 A g^−1^, which is much higher than the SWO-45 and SWO-75 electrodes. The excellent rate performance of SWO-60 could be associated with its unique nanosheets morphology, which facilitates rapid lithium-ion conduction and transportation [43].

**Figure 5:**
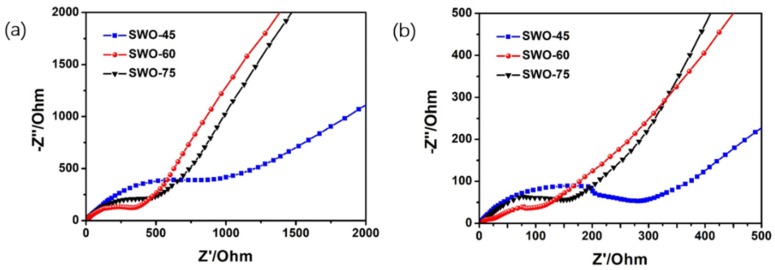
Nyquist plots of different electrodes (**a**) before cycling and (**b**) after 200 cycles at 0.2 A g^−1^.

In order to investigate the electrochemical kinetics of different kinds of electrodes, electrochemical impedance spectroscopy measurements were carried out from 0.1 Hz to 100 kHz in frequency. Figure 5a,b shows the Nyquist plots of three kinds of electrodes in initial their states and after 200 discharge–charge cycling at 0.2 A g^−1^, respectively. In the impedance spectra, the semicircles in the high-frequency region correspond to the charge-transfer resistance (*R*_ct_) in the interface of electrodes and electrolyte, and the inclined lines during the low-frequency region are attributed to insertion of lithium ions into the negative electrode [44]. In Figure 5a, the charge transfer resistance of a battery with a SWO-60 electrode (332.8 Ω) is much smaller than those of the SWO-45 (998.4 Ω) and SWO-75 (477.3 Ω) batteries, which is consistent with the good cycling stability of the SWO-60 electrode. Figure 5b shows the EIS of SWO-45, SWO-60, and SWO-75 cells after 200 cycles at 0.2 A g^−1^. After cycling, the cell with the SWO-60 electrode also exhibited the lowest charge transfer resistance, which is in good agreement with the fact that the SWO-60 electrode showed the best electrochemical properties.

**Figure 6:**
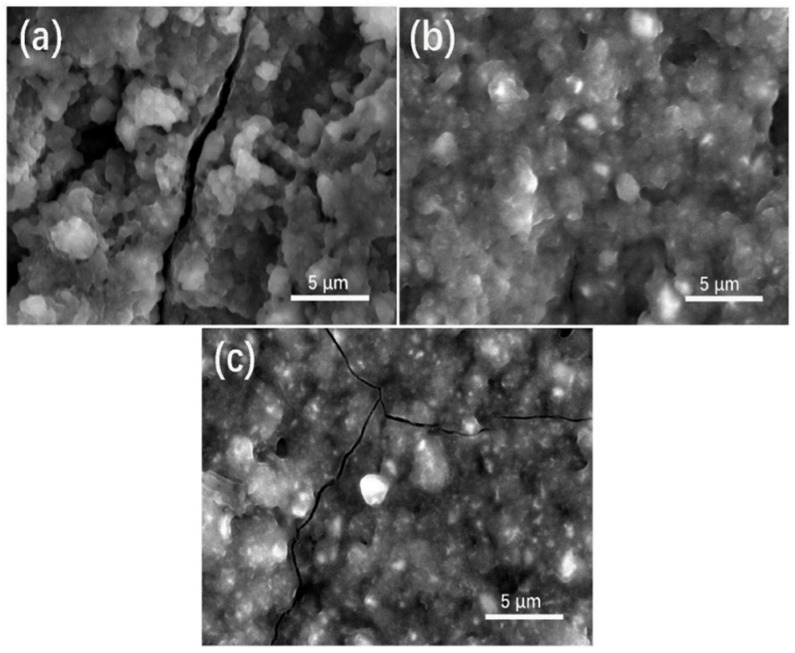
Scanning electron microscope images of (**a**) SWO-45, (**b**) SWO-60, and (**c**) SWO-75 electrodes after 200 cycles at 0.2 A g^−1^.

To investigate the effect of different microwave hydrothermal times on the structural stability of electrodes, the morphology of electrodes was examined after 200 cycles at 0.2 A g^−1^ (Figure 6). Figure 6a–c shows the morphology of the SWO-45, SWO-60, and SWO-75 electrodes after cycling, respectively. After 200 cycles, it was found that there were many large cracks and pulverizations in the SWO-45 and SWO-75 electrodes, as shown in Figure 6a,c, respectively. By contrast, the SWO-60 electrode retained its structural integrity even after 200 cycles, as shown in Figure 6b.

## 4. Conclusions

In summary, mesoporous Sb_2_WO_6_ nanosheets were successfully fabricated using a microwave hydrothermal method and were systematically investigated as negative electrodes for LIBs. When the microwave reaction time is 60 min, SWO-60 exhibits the highest reversible capacity and the best rate performance. The good electrochemical performance of the SWO-60 electrode may be ascribed to its uniformly distributed nanosheets structure. Due to the limited inherent electronic conductivity of SWO, our future work will focus on combining SWO with other materials with higher conductivities, such as heteroatom-doped nanocarbon materials and graphene, to further improve their electrochemical performance.

## Figures and Tables

**Table 1:** Comparison of Sb_2_WO_6_ samples synthesized by different microwave heating times in terms of specific surface area, mean pore size, and mean crystallite size.

Materials	Specific Surface Area (m^2^/g)	Mean Pore Size (nm)	Mean Crystallite Size (nm)
SWO-45	16.79	3.7	15.37
SWO-60	22.07	3.9	13.82
SWO-75	22.98	3.6	13.16
